# Mountain surface processes and regulation

**DOI:** 10.1038/s41598-021-84784-8

**Published:** 2021-03-05

**Authors:** Xuyang Lu

**Affiliations:** Institute of Mountain Hazards and Environment, Chinese Academy of Sciences, Chengdu, 610041 China

**Keywords:** Natural hazards, Ecology, Environmental impact

## Abstract

Mountains cover about a quarter of the world’s land surface, and directly support a significant proportion of the world’s population living within mountainous regions. Mountains provide water, timber and non-timber forest products, mineral resources, and many other food, fiber, and fuel products. Mountains also provide diverse ecosystems, in terms of both species and genetics, due to the topographic complexity in mountains increasing isolation and promoting speciation. Managing mountain regions for the sustainable delivery of critical goods and services requires an increasingly detailed understanding of mountain surface processes and regulation. The aim of this Guest Edited Collection is to provide a platform for interdisciplinary studies of mountain surface processes, and their responses to climate change and human activities.

Mountains are critical components of the Earth’s surface and provide vital resources, and essential ecosystems, to a significant proportion of the global population. As the water towers of the world, mountainous regions are important water suppliers for nearly half of all people^1^. In addition to water, mountains also yield timber and non-timber forest products, mineral resources, and many other food, fiber, and fuel products^2^. Moreover, mountain ecosystems provide carbon storage, nutrient retention, a provision of clean oxygen, a destination for tourism and recreation, pollinators, and pest control^3^. Therefore, mountainous regions guarantee ecosystem security, and are key natural resource bases for human development due to their ecological, social, and economic values^4^.

Mountains are widely distributed across the continents, from tropical to arctic latitudes, and are defined as landforms that rise prominently above their surroundings, generally exhibiting steep slopes, a relatively confined summit area, and considerable local relief^5^. Whilst there are three different approaches to mapping mountainous areas of the world, each with its own history and applications, it is clear that mountains comprise a significant proportion of global land area, from at least 12.4%, to as much as 30.5%^2,6–9^. Lithosphere dynamics, as well as related climate variability, create highly concentrated environmental gradients over short distances in mountainous regions, which cause environmental heterogeneity in terms of both biotic (land cover, vegetation, animals) and abiotic (climate, soil, topography) variables^5^, and for this reason, mountain systems can be particularly sensitive to climate and environmental changes^10,11^. For instance, recent studies show that temperature increases in mountains lead to the distribution ranges of mountain plant and animal species shifting upward along altitudinal gradients, which poses a risk of species extinction, if they are not able to adapt their life-cycles or migrate^12,13^.

Due to the combined impacts of both an increasing human population and climate change, there is an urgent need to sustainably manage mountain systems, in order to support human well-being. In addition, an increasingly detailed understanding of mountain system processes, and regulations in light of these pressures, is required. The aim of this Collection is to provide a platform for interdisciplinary studies of mountain surface processes, and their responses to climate change and human activities. The published papers in this Collection are classified into three subsections, mountain ecology, mountain environment, and mountain hazards. Some representative papers are highlighted as follows.

Mountain ecosystems are inhabited by highly specialised and endemic plants, animals, and microorganisms, which are particularly susceptible to climatic changes. Trant et al.^14^ measured the treeline advance of Canadian Rocky Mountain habitats with a time-lapse of 68 to 125 years and found that the treeline at higher altitudes and further north had a greater probability of advancing. Strinella et al.^15^ investigated how weather variables affect the white-winged snowfinch *Montifringilla nivalis* in the European Alps, and found that the low apparent survival may be due to recent global warming and weather, and that these variables affect males and females differently. Durán-Romero et al.^16^ found that bacterioplankton and phytoplankton in a high-mountain Mediterranean lake showed rapid and synergistic responses to combinations of global-change stressors. These responses suggest that bacterioplankton would better adapt to a future global change scenario than phytoplankton.

Mountain environment change could be affected and driven by geological processes, climate change, and human activity. Mair et al.^17^ quantified the long-term denudation rate of steep alpine headwalls by utilizing *in-situ* cosmogenic ^36^Cl depth profiles. They concluded that the denudation rate has been consistently high across the European Alps, over hundreds or even thousands of years. Liu et al.^18^ analyzed changes in the area of 14 lakes in Central Asia from 2001 to 2016. The results demonstrated that alpine lakes are trending towards expansion, and suggest this is due to the warmer and wetter climate. Spandre et al.^19^ simulated snow reliability in 129 ski resorts in the French Alps in the twenty-first century by using a dedicated snowpack model, and predicted that snow conditions would become frequently unreliable at 3 °C global warming and beyond, due to a marked drop in snow coverage with a mean frequency of snow-scarce seasons reaching 80%.

Natural hazards, including torrential floods, debris flows, landslides, and glacial lake outburst, often occur in mountain areas. Tichavský et al.^20^ found that dry spells and extreme precipitation were the main triggers of landslides in the Outer Western Carpathians of Central Europe, and Stoffel et al.^21^ found that small rockfalls near Monte Fitz Roy in Argentina, could be triggered by earthquakes of moderate intensity, at large distances from their epicenter. De Haas et al.^22^ identified a memory effect whereby erosion was stronger at locations where there had previously been strong deposition from debris flows. Erosion did not increase with debris-flow magnitude due to a limit of debris-flow bulking set by channel geometry. Yousefi et al.^23^ developed a machine learning framework for multi-hazards modeling and presented a multi-hazard risk map for five natural hazards (floods, landslides, land subsidence, snow avalanches, and forest fires) in southwestern Iran.

The Collection was published in October 2020, however submissions are still being welcomed on a rolling basis. Papers accepted will then be added to the Collection as and when they are published. We would like to thank all the authors and reviewers who have contributed to this Collection. Even though this Collection is just the tip of the iceberg in terms of the numerous mountain studies, we firmly believe that our efforts help to promote an understanding of mountain surface and regulation, as well as its response to climate change and human activity.
